# Dr. Harry B. Howell

**Published:** 1914-04

**Authors:** 


Dr. Harry B. Howell, Buffalo, 1890, died in Rochester, February 6, aged 50.

